# Animal welfare and society—Part 4, Response to Gary Francione

**DOI:** 10.1093/af/vfac008

**Published:** 2022-03-17

**Authors:** Jocelyne Porcher

**Affiliations:** UMR Innovation, Montpellier, France

I see two main points of agreement with Gary Francione (GF): 1) “animal welfare” does not change anything substantial for the farm animals themselves and 2) vegetarianism is a moral aporia. The individual and collective solution to bringing domestic animals out of their condition is, therefore, not to become vegetarians. I will not discuss here the fact that veganism cannot be considered, and indeed is not by vegan associations, as a universal practical solution. Rather, it is cellular agriculture, of which People for the Ethical Treatment of Animals (PETA) and historical “animal cause” theorists like Peter Singer are active supporters, which is advanced as the only alternative to industrial violence against animals. My experience of working with animals as an animal farmer, my 25 yr of research on animal husbandry working with farmers and their animals, and a life lived in the company of animals may explain two fundamental points of divergence with the reasoning of GF. I will detail them below: 1) the concealment of the notion of work in the analysis of our relations with animals and 2) the assimilation between the worlds of animals and of humans.

## 1) The Concealment of the Notion of Work

By focusing our relationships with animals on the issue of property, GF obscures the issue of work, which is at the heart of our relationships with domestic animals. We do not live with animals *next to* each other. Rather, we live together and we work together. As a reminder, to work, as occupational psychology has shown, is not only to produce, but it is also to live together and to perform. In other words, work has not only productive rationalities but also, and above all, relational, identity, moral, and aesthetic rationalities, among others. As my research has shown, in animal husbandry, the primary rationality is relational rationality. We work with animals because we want to live with them and they with us.

By ignoring work, and therefore depoliticizing our relationships with animals, GF erases the historical and social springs that allow us to understand them. Such reasoning erases the historical distinctions between animal husbandry and animal production and the fact that animal production is built *against* animal husbandry. From my point of view, the 19th century is not one of the rise of sensitivity toward animals; on the contrary, it is one of the industrial organization of animal exploitation in the context of the emergence of industrial capitalism; exploitation carried by the bourgeoisie (upper class), scientists, and industrialists, accompanied by its moral compassionate counterpart. Simply recall that the first animal welfare associations were born in England at the same time as industrial society was developing and that violence against humans and animals was crystallized in factories and in cities, well described by Dickens (e.g., in “Hard Times”). In France, the bourgeois voting the Grammont law, which penalizes public brutality toward animals, were also shareholders of the mines where children and horses toiled and suffered far from the circles where, between men, laws were discussed.

The question of work is central to understanding our relationship with domestic animals. From this perspective, representing the reality of our relationships, there is no difference between a dog and a cow. Both are at work with us. The first provides a service, for example, company and the other foodstuffs. Their work involves different working conditions and working life. Thus, the companion dog lives alone or with a fellow creature with his master. The purpose of dog work is not to provide income for the owner. On the contrary, the master’s income finances the relationship. The companion dog is essentially immersed in a human world where, for him, emotional and relational work is paramount. Keeping company for a dog is indeed not natural. It is work. It is a question of “keeping company,” that is to say of being present, attentive, at the service of his master, obedient, and affectionate among others. The majority of dog professions (e.g., assistance, police, or army) involve a period of training, in contrast to companion dogs. This absence of training may lead to difficult relationships well known to veterinarians, sometimes resulting in the euthanasia of the dog.

A cow does not live alone with her breeder. She lives in a herd, on the one hand, because her production is often part of a collective production, necessary to ensure an income for the farmer and, on the other hand, because cows are social animals and animal husbandry respects this sociability. The big difference between cows and dogs is that the dog does not go to the slaughterhouse (although often he finds his end at the veterinary clinic). The obvious reason is that the work of the “companion” dog, on the one hand, does not require generating income and, on the other, does not require that room is made for younger counterparts. In husbandry, on the contrary, for animals to be born, other animals must leave. A husbandry system is a finite system. If no animal came out of it, it would be saturated and lead to starvation. Animal shelters present similar characteristics. As there is no official animal work, the shelter must find income or function as a zoo, that is to say, charge for admission to the shelter, which is often the case. The work of animals is then, as in the zoo, one of representation and relationships. In a shelter too, if you want to accept animal entrances, others must leave. The resources that a system can offer are limited.

The concealment of the work of “companion” animals leads to morally legitimizing their presence as if it was not part of the ties that bind us to domestic animals. Adopting a dog is de facto assuming asymmetrical working relationships. It is fortunate for vegans that people abandon their dogs; otherwise, they would be forced to follow their breakup logic to the end and actually live *without* animals.

## 2) The Assimilation between Animal and Human Worlds

I find it particularly condescending for animals to think of them as humans, that is, to compare them to children or to imagine that their perceived world is similar to ours. Animal worlds are different from each other and from ours, and it is only in our common workspace that we can build common references. The world of cows is not like ours and it has intrinsic values beyond what we can imagine. Cows do not see the past, future, or death the same way we do. We must respect their specificity and not think only in reference to ourselves. Not only cows and pigs but also dogs and horses have specific relationships with the world that we must envision outside of our own representations of good or evil. It is by living and working with animals that we perceive this otherness. I do not like cows, pigs, or dogs because I consider them to be neighbors but precisely because they are not. They are different from me and different from each other. They have different languages, values, and behaviors. They do not speak. They do not write books about the meaning of life. Nevertheless, they have many other ways of understanding the world, of making it understood, and of sharing it. All these differences make our relationship rich. When an outdoor pig farmer slaughters a pig in the meadow in the middle of the herd, the other pigs do not rebel, run away, or exclaim “oh my god!”. They take note of their fellow’s immobility and return to their occupations. Death is part of their life—of their life with us and of their life without us. What matters to them from the point of view of our domestic relationships, whether it is a dog or a cow, is the meaning and the beauty of life with us, the happy times they were able to experience and to share with us.

For my part, I have a huge admiration for domestic animals. They taught me a thousand things that I would not have learned without them—on life, death, work, and nature. My dog showed me, at my expense, how seriously he took his work as a sheepdog, how involved in the work he was, and how eager he was to do a good job with the sheep. The sheep taught me patience and responsibility and helped me to find my place somewhere between the earth below my feet and the horizon. The pigs instilled in me sincerity, resilience, and pity. All of them helped me to understand the relationships between dependence and freedom, pleasure and pain, and ignorance and pride. By putting my life at their service, my life as a farmer and my life as a researcher, like others, I believe that I have lived up to them and have kept to them the promises of work and the exigency of giving.

## About the Author

Jocelyne Porcher is a zootechnician and sociologist, research director at INRAE (PhD in zootechnie, HDR in sociology). Before becoming a researcher, she was an animal farmer, an employee in industrial pig production, and a technician in organic farming. Her research focuses on the working relationship between humans and animals. She is the author of a dozen books on the subject. She is particularly interested in the affective relations between humans and animals in the workplace, in suffering and pleasure at work, in the slaughter of animals and alternatives to industrial slaughter, in animal labor, and in cellular agriculture. She also produces a critical sociology of the “animal cause” and its logic of development within the capitalist system.

